# Anterior cingulate glutamate metabolites as a predictor of antipsychotic response in first episode psychosis: data from the STRATA collaboration

**DOI:** 10.1038/s41386-022-01508-w

**Published:** 2022-12-01

**Authors:** Alice Egerton, Kira Griffiths, Cecila Casetta, Bill Deakin, Richard Drake, Oliver D. Howes, Laura Kassoumeri, Sobia Khan, Steve Lankshear, Jane Lees, Shon Lewis, Elena Mikulskaya, Edward Millgate, Ebenezer Oloyede, Rebecca Pollard, Nathalie Rich, Aviv Segev, Kyra-Verena Sendt, James H. MacCabe

**Affiliations:** 1 Department of Psychosis Studies, Institute of Psychiatry, Psychology & Neuroscience, King’s College London, London, UK; 2 NIHR Biomedical Research Centre at South London and Maudsley NHS Foundation Trust, London, UK; 3 Division of Neuroscience and Experimental Psychology, School of Biological Sciences, Faculty of Biology, Medicine and Health, University of Manchester, Manchester, UK; 4 Greater Manchester Mental Health NHS Foundation Trust Bury New Road, Prestwich, Manchester M25 3BL UK; 5 Division of Psychology and Mental Health, School of Biological Sciences, Faculty of Biology, Medicine and Health, University of Manchester, Manchester, UK

**Keywords:** Predictive markers, Neurotransmitters, Psychosis, Schizophrenia

## Abstract

Elevated brain glutamate has been implicated in non-response to antipsychotic medication in schizophrenia. Biomarkers that can accurately predict antipsychotic non-response from the first episode of psychosis (FEP) could allow stratification of patients; for example, patients predicted not to respond to standard antipsychotics could be fast-tracked to clozapine. Using proton magnetic resonance spectroscopy (^1^H-MRS), we examined the ability of glutamate and Glx (glutamate plus glutamine) in the anterior cingulate cortex (ACC) and caudate to predict response to antipsychotic treatment. A total of 89 minimally medicated patients with FEP not meeting symptomatic criteria for remission were recruited across two study sites. ^1^H-MRS and clinical data were acquired at baseline, 2 and 6 weeks. Response was defined as >20% reduction in Positive and Negative Syndrome Scale (PANSS) Total score from baseline to 6 weeks. In the ACC, baseline glutamate and Glx were higher in Non-Responders and significantly predicted response (*P* < 0.02; *n* = 42). Overall accuracy was greatest for ACC Glx (69%) and increased to 75% when symptom severity at baseline was included in the model. Glutamate metabolites in the caudate were not associated with response, and there was no significant change in glutamate metabolites over time in either region. These results add to the evidence linking elevations in ACC glutamate metabolites to a poor antipsychotic response. They indicate that glutamate may have utility in predicting response during early treatment of first episode psychosis. Improvements in accuracy may be made by combining glutamate measures with other response biomarkers.

## Introduction

Although antipsychotic medication can be highly effective in reducing the severity of symptoms of schizophrenia, there is significant variation in the degree to which symptoms will improve with treatment [1, 2]. In approximately one third of patients, symptoms will not respond adequately to sequential treatment with non-clozapine antipsychotics and this subgroup are defined as meeting criteria for treatment resistant schizophrenia (TRS), for which the associated recommendation is to initiate treatment with clozapine [3, 4]. In a high proportion of TRS cases, a poor antipsychotic response may be present from illness onset [5], however currently TRS can only be identified retrospectively through failed treatment trials [4]. Furthermore, TRS is typically not identified until several years after TRS criteria have been met, so the initiation of clozapine is delayed, and during this time patients undergo ineffective treatment and prolonged duration of active symptoms. These delays in initiating adequate treatment are associated with poorer clinical outcomes [3, 6, 7]. Biomarkers that can accurately predict antipsychotic non-response from early after presentation with first episode psychosis (FEP) could aid clinical decisions to initiate clozapine earlier, and thereby greatly improve health and economic outcomes [8]. In addition, elucidating the neurobiology of non-response may lead to therapeutic targets for this group.

Brain glutamate is one potential biomarker of antipsychotic non-response [9]. Cross-sectional studies measuring brain glutamate using proton magnetic resonance spectroscopy (^1^H-MRS) have shown that non-remission of symptoms in FEP [10], TRS and clozapine-resistant schizophrenia [11–15] are associated with greater concentrations of glutamate metabolites in the medial frontal cortex (MFC)/anterior cingulate cortex (ACC). Meta-analysis has identified increased MFC/ACC glutamate metabolites in the TRS illness subtype [16] and mega-analysis of individual data from multiple studies in schizophrenia has shown that higher ACC glutamate metabolites are associated with more severe total and positive symptoms and worse functioning [17]. A previous prospective study found that ACC glutamate levels in minimally medicated FEP predicted subsequent non-remission following 4 weeks of treatment with amisulpride with 69% overall accuracy, increasing to 75% when age and baseline symptom severity were included in the model [18]. Other prospective studies in FEP have found that non-response is associated with higher glutamate metabolite concentrations in the striatum [19] or thalamus [20, 21]. Glutamate elevation may therefore contribute to poor treatment outcomes in schizophrenia, and this may be present from FEP. Accordingly, ^1^H-MRS glutamate metabolites could be a biomarker to predict treatment outcomes, which could be applied in the first weeks after presentation to aid clinical decision-making. Previous prospective studies examined glutamate in participants who were antipsychotic naïve or minimally medicated (<2 weeks) at baseline as a predictor of response to a single antipsychotic compound [18–20, 22]. It is not known whether these findings will generalise to the more naturalistic conditions of being measured within the first weeks of standard antipsychotic treatment.

Treatment with antipsychotic medication has been associated with reductions in glutamate in longitudinal studies [23, 24], including in the MFC/ACC [18, 25] and right caudate [26–28]. This hypothesis is supported by mega-analysis showing that higher doses of antipsychotics are associated with lower MFC glutamate concentrations [17]. Some studies have observed correlations between decreases in glutamate metabolites and symptomatic improvement [21, 23, 26, 28]. However, the elevation in MFC/ACC glutamate in patients who have not responded to equivalent or higher doses of antipsychotic medication [10–15] may indicate that reductions in glutamate metabolites on antipsychotic treatment are more likely to occur in treatment responsive illness. Potentially, early glutamatergic changes during initial antipsychotic treatment might better predict later clinical outcome, as has been suggested for changes in brain activity [29–31], but this has not yet been investigated.

In this observational study we examined glutamate metabolites in the ACC and striatum in a FEP cohort undergoing antipsychotic treatment over 6 weeks, across two study sites. Our primary hypothesis was that elevated glutamate metabolites at baseline would be associated with a poorer response to antipsychotic treatment. We also hypothesised that glutamate metabolites would be reduced during antipsychotic treatment, and that early reductions in glutamate metabolites over 2 weeks would be associated with a better clinical outcome at 6 weeks.

## Methods

### Participants

Data were collected as part of the ‘STRATA-2’ study, examining the biological effects of antipsychotic treatment, which had National Health Service Research Ethics Approval (Reference 17/NI/0209). Participants were recruited and assessed across two U.K. sites, King’s College London (KCL) and the University of Manchester (UoM). Patients gave written informed consent to participate. The study was open to patients 18–65 years of age, who were able to read and write in English at a level suitable to complete study procedures. Inclusion also required a DSM-5 diagnosis for schizophrenia, schizoaffective disorder, schizophreniform disorder or psychosis non-specified; being within the first 2 years of onset of the psychotic illness; minimal previous treatment with antipsychotic medication, defined as having received antipsychotic treatment for no longer than 4 weeks, after a period of being either antipsychotic naïve or antipsychotic-free for at least 14 days; and that at screening patients did not meet symptomatic criteria for remission [32]. Exclusion criteria included pregnancy or breastfeeding; meeting ICD-10 criteria for harmful substance misuse, psychotic disorder secondary to substance misuse or an organic brain disorder; treatment with clozapine in the last 3 months; history of severe head injury involving loss of consciousness for >5 min; the presence of standard contraindications to MRI at 3 Tesla or severe claustrophobia prohibiting MRI participation.

### Study overview

The study included an initial screening visit followed by 3 assessment visits. The assessment visits were conducted within 4 weeks of commencing antipsychotic treatment (‘baseline’) and 2 and 6 weeks (each ± 7days) after baseline. Each assessment visit included MRI acquisition and clinical interview. Throughout the study participants continued to take antipsychotic medication as according to their normal clinical care.

### Clinical and demographic interview

During the screening visit, following provision of informed consent, demographic information and medical history were recorded and the Mini International Neuropsychiatric Interview (MINI) was used to confirm diagnosis. At the screening and each assessment visit, symptom severity was evaluated using the Positive and Negative Syndrome Scale for Schizophrenia (PANSS) [33], illness severity was evaluated using the Clinical Global Impression - Severity scale for schizophrenia (CGI-S), details of all prescription and non-prescription medications were recorded and adherence with antipsychotic medication was evaluated using the Clinician Rating Scale (CRS) [34].

### ^1^H-Magnetic Resonance Spectroscopy

Data were acquired at 3 Tesla on either a General Electric (GE) MR750 (General Electric Healthcare, Chicago, USA; KCL site) or a Philips Achieva (Philips Healthcare, The Netherlands; UoM site) magnetic resonance scanner. Following localiser and calibration sequences, sagittal T1-weighted (T1-w) accelerated ADNI-GO sequences were acquired as according to the details on the ADNI website http://adni.loni.usc.edu/methods/documents/mri-protocols/. T1-w images were automatically reformatted to provide axial, sagittal and coronal views. Voxel positioning was planned on the axial T1-w image and checked across all orientations. The centre of the 20 × 20 × 20 mm ACC voxel was positioned on the midline sagittal localizer 16 mm above the most anterior portion of the genus of the corpus callosum, with the most posterior edge of the voxel approximately 2 mm from the edge of the corpus callosum. The 20 × 20 × 20 mm right caudate voxel was positioned to include the maximal amount of caudate grey matter, and with the lower edge 3 mm dorsal to the anterior commissure [12]. Voxel positioning and an example spectrum from each site is shown in Supplementary Figs. 1 and 2. ^1^H-MRS data were acquired using Point RESolved Spectroscopy (PRESS), at a TE = 35 ms, TR = 2000 ms, 128 averages, bandwidth/sample frequency of ± 5000 Hz, 4096 complex points. On the GE system data were acquired using the standard GE PROton Brain Examination (PROBE) sequence. Unsuppressed water spectra were acquired in the same voxel locations. ^1^H-MRS details are provided in the minimum reporting standards for in vivo magnetic resonance spectroscopy checklist (Supplementary Table 1), according to consensus recommendations [35].

### ^1^H-MRS data processing and quality control

All imaging data were transferred to KCL for analysis. ^1^H-MRS spectra were analysed using LCModel version 6.3–1M [36] using the standard LCModel basis set, acquired using PRESS at 3 Tesla and 35 ms, and containing 16 metabolites (L-alanine, aspartate, creatine, phosphocreatine, GABA, glucose, glutamine, glutamate, glycerophosphocholine, glycine, myo-inositol, L-lactate, N-acetylaspartate, N- acetylaspartylglutamate, phosphocholine, taurine). All metabolite estimates were water referenced. Gannet version 3.0 (http://gabamrs.com) was used to co-register the voxels with the corresponding T1-w image to determine the voxel tissue fractions. Metabolite estimates (M) were corrected for the voxel fractions of white matter (WM), grey matter (GM) and cerebrospinal fluid (CSF) using the equation M_corr_ = M(WM + 1.21GM + 1.55CSF)/(WM + GM) [37, 38]. The primary metabolite of interest was glutamate, reported here both as Glu_corr_, and as the summed signal of glutamate and glutamine (Glx_corr_) in the ACC and in the caudate. Quality control for spectra followed our previously described procedure [12]. Individual metabolite estimates associated with Cramer Rao Lower Bounds (CRLB) > 20% were excluded.

### Statistical analysis

To account for site effects, baseline ^1^H-MRS data were converted to z-scores prior to analyses, by subtracting the site mean from individual values before dividing by the site standard deviation using the data acquired in this study. Potential effects of demographic or clinical variables were investigated using *t-*tests or Pearson’s correlation analyses. Percentage reduction in PANSS total score was calculated after subtraction of minimum possible scores [39]. Responders were defined as patients who showed a > 20% reduction in PANSS total score from baseline to 6 weeks, and non-responders as a reduction of 20% or less. This aligns with the consensus recommendations of <20% symptom reduction over ≥6 weeks for identifying antipsychotic non-response during prospective treatment periods [4]. Binary logistic regression determined whether Glu_corr_ and Glx_corr_ at baseline predicted response status at 6 weeks. The accuracy of significant prediction was evaluated by estimating the area under the receiver-operating curve (ROC). As percentage symptom reduction early into antipsychotic treatment can predict longer-term antipsychotic response [40–42] the percentage reduction in PANSS total score from baseline to 2 weeks was subsequently added to the model. Linear regression determined continuous relationships between baseline Glu_corr_ and Glx_corr_ and the change in PANSS total score over 6 weeks, covarying for PANSS total score at baseline. Where results for PANSS total score were significant, the relationships with subscale scores were subsequently evaluated to determine the contributing symptom domains. Effects of time and 6-week response status on Glu_corr_ and Glx_corr_ over the 3 assessment visits was examined using linear mixed effects modelling, accounting for random effects of site. For all analyses, the threshold for statistical significance was corrected to α = 0.025 to account for the two voxels of interest. Significance was not adjusted for the two glutamate measures (Glu_corr_ and Glx_corr_) as these are non-independent. Analyses were performed in SPSS version 27.

## Results

A total of 89 patients were recruited to the study (Supplementary Fig. 3). Of these, 45 completed at least one MRI scan and the clinical assessments through to the 6-week follow-up (Table 1). Ten of these 45 participants had received previous antipsychotic medication; the reasons for cessation included adverse effects (*n* = 5), non-adherence (*n* = 4), or were unknown (*n* = 1). At 6 weeks, 27 patients were classified as responders and 18 were classified as non-responders. These groups did not differ in any clinical or demographic characteristics at baseline, or in antipsychotic chlorpromazine equivalents (CPZE) or adherence score on the CRS at 6 weeks (Table 1). Percentage reduction in PANSS total score from baseline to 2 weeks was associated with response status at 6 weeks (B = −0.03; S.E. = 0.01; Wald = 3.77; df = 1; P = 0.05; Exp(B) = 0.98).

**Table 1:** Demographic and clinical characteristics of participants who completed 6-week follow-up.

	Total sample	Responders	Non-Responders
***n***	45	27	18
Sex. *n* male (%)	33 (73%)	18 (67%)	15 (83%)
Age, years	27.53 ± 8.36	26.30 ± 7.88	29.39 ± 8.93
^a^Duration of psychosis, months	12.76 ± 21.56	15.85 ± 26.79	8.11 ± 8.20
**Number of hospitalisations,** ***n***			
None	14	10	4
One	25	14	11
Two	5	3	2
Three	1	0	1
**Number of previous antipsychotic trials,** ***n***			
None	35	22	13
One	8	4	4
Two	2	1	1
^b^Days on antipsychotic medication	20.02 ± 6.47	19.20 ± 6.49	21.24 ± 6.45
**Prescribed antipsychotic at baseline,** ***n***			
Aripiprazole	19	13	6
Olanzapine	17	9	8
Risperidone	3	2	1
Quetiapine	4	2	2
Amisulpride	1	0	1
Paliperidone	1	1	0
CPZE/mg/day at baseline	176.33 ± 123.31	154.63 ± 65.06	208.89 ± 176.10
CPZE/mg/day at 6 weeks	182.75 ± 96.30	172.00 ± 81.43	200.67 ± 117.97
Adherence at baseline	5.53 ± 1.34	5.59 ± 1.58	5.44 ± 0.92
Adherence at 6 weeks	5.04 ± 2.09	5.41 ± 1.93	4.50 ± 2.26
**Symptom Severity at Baseline**			
PANSS Total	73.07 ± 15.86	73.96 ± 15.74	71.22 ± 16.39
PANSS Positive	18.91 ± 4.74	18.96 ± 4.46	18.83 ± 5.27
PANSS Negative	17.67 ± 6.71	17.67 ± 6.39	17.67 ± 7.35
PANSS General	36.49 ± 8.41	37.33 ± 8.36	35.22 ± 8.57
CGI-S	4.51 ± 1.06	4.63 ± 1.01	4.33 ± 1.14
**Symptom Severity at 2 weeks**			
PANSS Total	66.92 ± 19.80	61.64 ± 13.16	76.36 ± 26.01*
PANSS Positive	16.05 ± 5.40	15.12 ± 3.54	17.71 ± 7.58
PANSS Negative	17.54 ± 6.82	16.40 ± 5.92	19.57 ± 8.04
PANSS General	33.33 ± 10.53	30.12 ± 7.10	39.07 ± 13.26*
CGI-S	4.00 ± 1.18	3.74 ± 0.96	4.43 ± 1.40
**Symptom Severity at 6 weeks**			
PANSS Total	61.36 ± 19.10	52.48 ± 12.00	74.67 ± 20.29*
PANSS Positive	14.13 ± 4.96	12.30 ± 3.48	16.89 ± 5.62*
PANSS Negative	16.00 ± 7.45	13.70 ± 5.89	19.44 ± 8.34*
PANSS General	31.22 ± 9.65	26.48 ± 5.89	38.33 ± 9.93*
CGI-S	3.18 ± 1.11	2.85 ± 0.88	3.67 ± 1.24*
**% Change in Symptom Severity Baseline to 6 weeks**.			
PANSS Total	−26.86 ± 32.57	−49.25 ± 16.15	6.72 ± 18.88
PANSS Positive	−37.15 ± 44.26	−55.71 ± 23.42	−9.32 ± 53.52
PANSS Negative	−13.63 ± 50.00	−35.70 ± 44.57	19.47 ± 38.70
PANSS General	−20.05 ± 50.22	−49.93 ± 20.91	24.76 ± 48.12
**Substance use at baseline**			
Cigarette smoking (unknown/none/less than daily/daily)	1/22/4/15	0/15/1/10	1/7/3/5
Alcohol current, Yes (%)	17 (38%)	8 (30%)	9 (50%)
Cannabis current, Yes (%)	8 (18%)	5 (19%)	3 (17%)
Other substance use, Yes (%)	9 (20%)	5 (19%)	4 (22%)

Data are presented as mean ± standard deviation unless otherwise specified.

^a^ Duration of psychosis defined as number of months between onset of first psychotic symptom and date of consent.

^b^Days on antipsychotic medication was defined as the number of days between start date of antipsychotic medication and the baseline MRI scan. *CGI-S* Clinical Global Impression Severity scale, *CPZE* chlorpromazine equivalent dose, *PANSS* Positive and Negative Syndrome Scale, *n* sample size.

*Significant difference between Responders and Non-Responders, *P* < 0.05.

### ^1^H-MRS data quality

MRI data were acquired in 53 patients at baseline, 38 patients at 2 weeks, and 39 patients at 6 weeks. 33 patients completed MRI at all 3 timepoints. ACC spectra were available in all scans acquired at each time-point. Caudate spectra were available in 45 of the 53 baseline scans, 36 of the 38 2-week scans, and 36 of the 39 6-week scans. Metabolite concentrations and spectral quality measures at each time-point are provided in Supplementary Tables 2 and 3. Baseline Glu_corr_ and Glx_corr_ concentrations were normally distributed and did not differ by sex, age, or smoking (non-smoker vs daily smoker), alcohol, cannabis, other substance use, duration of psychosis nor antipsychotic CPZE at baseline (all *P* > 0.05).

### Baseline glutamate metabolites and prediction of response

**Fig. 1:**
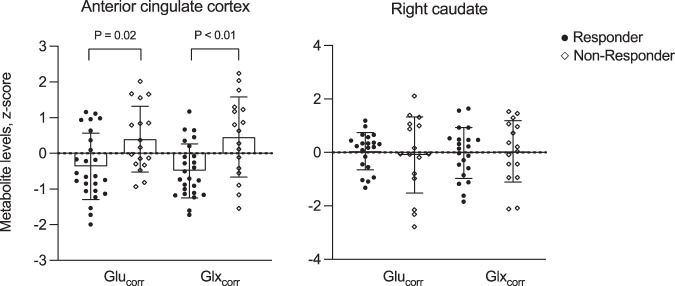
Glutamate metabolite levels in the anterior cingulate cortex and right caudate at baseline in patients classified as Responders or Non-Responders at 6 weeks. Glu glutamate, Glx glutamate + glutamine. The graphs present the individual values, mean and standard deviations.

For the primary hypotheses testing the association between baseline glutamate metabolites and response at 6 weeks, data from 42 individuals were available for the ACC and 37 were available for the caudate. Both ACC Glu_corr_ and Glx_corr_ at baseline significantly predicted response status at 6 weeks (Glu_corr_ B = 0.88; S.E. = 0.38; Wald = 5.47; df = 1; *P* = 0.02; Exp(B) = 2.42; Glx_corr_: B = 1.07; S.E. = 0.40; Wald = 7.33; df = 1; *P* < 0.01; Exp(B) = 2.92). This was related to increased concentrations of ACC Glu_corr_ and Glx_corr_ in the Non-Responder compared to Responder group, with effect sizes of *d* = 0.82 and 1.02 respectively (Glu_corr_ t_40_ = −2.62; *P* = 0.012; Glx_corr_ t_40_ = −3.27; *P* = 0.002, Table 2, Fig. 1). ROC analysis returned an area under curve of 0.72 for Glu_corr_ and 0.75 for Glx_corr_. Glu_corr_ was associated with an overall accuracy of 62%, sensitivity of 66% (proportion of Responders correctly predicted to respond on the basis of baseline Glu_corr_), specificity 54% (proportion of Non-Responders correctly predicted not to respond on the basis of baseline Glu_corr_), positive predictive value (PPV) of 76% and a negative predictive value (NPV) of 41%. Glx_corr_ was associated with an overall accuracy of 69%, sensitivity 71%, specificity 64%, PPV 80% and NPV 53%. Including both ACC Glx_corr_ and early percentage change in PANSS Total score in the model increased the overall accuracy to 75%, sensitivity to 79%, specificity to 67%, PPV to 82% and NPV to 62%. The associations between baseline ACC Glu_corr_ and Glx_corr_ and response status remained significant when covarying for antipsychotic CPZE dose or CRS scores at 6 weeks (*P* = 0.01 to 0.04). Caudate Glu_corr_ and Glx_corr_ at baseline was not significantly associated with response status (Glu_corr_ B = −0.13; S.E. = 0.32; Wald = 1.61; df = 1; *P* = 0.68; Exp(B) = 0.88; Glx_corr_: B = 0.06; S.E. = 0.33; Wald = 0.03; df = 1; *P* = 0.87; Exp(B) = 1.06, Table 2, Fig. 1). None of the other metabolites showed significant associations with response (threshold *P* < 0.025, Table 2).

**Table 2:** ^1^H-MRS metabolite estimates at baseline in the Responder and Non-Responder groups at 6 weeks.

	Responder	Non-responder
**Anterior cingulate**		
Sample size	25	17
Glutamate	−0.36 ± 0.93	0.40 ± 0.92**
Glx	−0.49 ± 0.76	0.46 ± 0.70**
NAA	−0.26 ± 0.87	0.39 ± 1.04
Choline	−0.17 ± 0.78	0.25 ± 1.23
Myo-Inositol	−0.40 ± 0.75	0.32 ± 1.28
Creatine	−0.23 ± 0.97	0.35 ± 0.93
**Right Caudate**		
Sample size	21	16
Glutamate	0.05 ± 0.70	−0.91 ± 1.42
Glx	−0.02 ± 0.95	0.04 ± 1.15
NAA	−0.08 ± 0.91	−0.08 ± 0.91
Choline	−0.23 ± 0.35	0.35 ± 1.56
Myo-Inositol	−0.05 ± 1.04	−0.08 ± 0.96
Creatine	−0.05 ± 0.83	−0.22 ± 1.20

Data are presented as mean ± standard deviation. Values represent z scores. *Glx* glutamate plus glutamine; *NAA* N-acetylaspartate plus N-acetylaspartylglutamate. *P < 0.025.

### Baseline glutamate metabolites and continuous measures of response

**Fig. 2:**
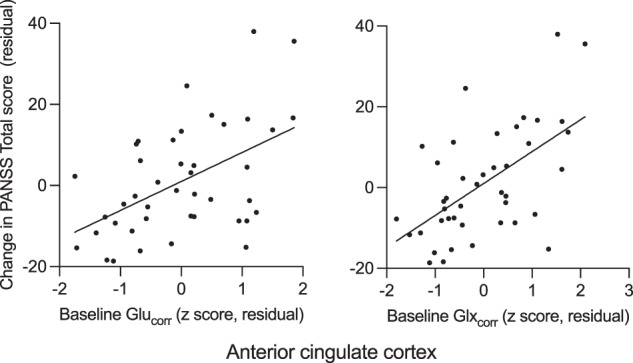
Association between glutamate metabolite levels in the anterior cingulate cortex at baseline and change in PANSS total score from baseline to 6 weeks, covaring for PANSS total score at baseline. Positive values for the change in PANSS represent increases in symptom severity. Glu_corr_ glutamate, Glx_corr_ glutamate + glutamine, PANSS positive and negative syndrome scale for schizophrenia. The relationships between both ACC Glu_corr_ and Glx_corr_ at baseline and change in PANSS Total score over 6 weeks were significant, *P* < 0.001.

ACC Glu_corr_ and Glx_corr_ at baseline showed positive associations with the change in symptom severity over 6 weeks, such that lower levels of glutamate metabolites at baseline were associated with greater improvements in symptom severity over time (Fig. 2). There was a significant relationship between baseline ACC Glu_corr_ and Glx_corr_ and change in PANSS Total score (Glu_corr_ Beta = 0.50; *t* = 3.66; *P* < 0.001; Glx_corr_ Beta = 0.43; *t* = 4.39; *P* < 0.001) and this was also apparent across the majority of the PANSS symptom subscales (ACC Glu_corr:_ PANSS Positive: Beta = 0.44; *t* = 3.51; *P* = 0.001; PANSS Negative: Beta = 0.18; *t* = 1.82; *P* = 0.08; PANSS General: Beta = 0.41; *t* = 3.39; *P* = 0.02; ACC Glx_corr_ PANSS Positive: Beta = 0.58; *t* = 3.97; *P* < 0.001; PANSS Negative: Beta = 0.25; *t* = 2.57; *P* = 0.01; PANSS General: Beta = 0.43; *t* = 3.72; *P* < 0.001). Covarying for CPZE dose or CRS at 6 weeks did not meaningfully alter these relationships. In the caudate, Glu_corr_ and Glx_corr_ at baseline were not significantly associated with the change PANSS total scores over 6 weeks (*P* = 0.21 and 0.36). ACC and caudate Glu_corr_ and Glx_corr_ at baseline were not significantly associated with PANSS Total score at baseline (*P* = 0.09 to 0.57).

### Changes in glutamate metabolites over 6 weeks

**Fig. 3:**
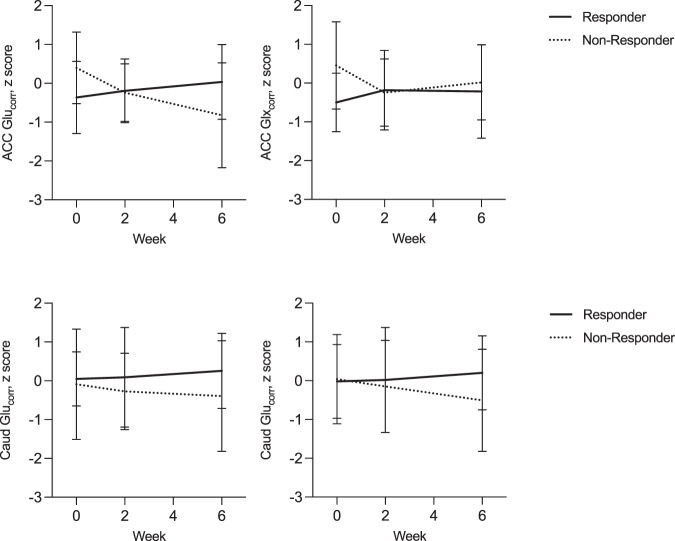
Glutamate metabolite levels in the anterior cingulate cortex (ACC) and right caudate (Caud) at baseline, 2 and 6 weeks in patients classified as Responders or Non-Responders at 6 weeks. Glu_corr_ glutamate, Glx_corr_ glutamate + glutamine. The graphs present the mean and standard deviations. All main effects of time, response, and response-by-time interactions were non-significant.

There were no significant changes in glutamate metabolite concentrations over time, and the effect of time on glutamate metabolites also did not differ according to response status (measured at 6-weeks) (Supplementary Table 4; Fig. 3). There were no significant relationships between the symmetrized percentage change in glutamate metabolites over 2 or 6 weeks and response status or change in PANSS Total score at 6 weeks (all *P* > 0.025).

## Discussion

In this observational, longitudinal study we examined glutamate metabolites in the ACC and striatum in a cohort of patients with FEP undergoing antipsychotic treatment according to their normal clinical care. The main finding was that higher levels of glutamate metabolites in the ACC were predictive of non-response to antipsychotic treatment, supporting our primary hypothesis. ACC glutamate metabolite levels at baseline were predictive of improvements across positive, negative, and general symptom domains. In contrast, glutamate metabolites in the caudate were not associated with response. We did not detect significant reductions in glutamate metabolites over time, nor associations between early glutamatergic change over 2 weeks and symptom severity at 6 weeks. Our results support the view that ACC glutamate elevations are associated with a poor antipsychotic response. With gains in predictive accuracy, ACC glutamate measures could form one component of a multivariate model to predict antipsychotic response from the first episode of psychosis.

The finding that elevated ACC glutamate metabolites are linked to a subsequently poor antipsychotic response is consistent with our previous prospective study examining FEP [18]. It is also accordant with the findings from most cross-sectional studies [10–16, 43] and meta-analysis [16] showing elevated ACC glutamate metabolites in antipsychotic nonresponsive patient groups. In contrast, two longitudinal studies in FEP have not found an association between elevated ACC glutamate metabolites at baseline and subsequent non-remission [20, 44] and one found the opposite relationship [22]. These studies examined antipsychotic naïve participants and one possibility is that antipsychotic exposure prior to baseline in our current and previous [18] cohorts may have elicited changes in glutamate, and this effect could potentially relate to associations with subsequent response. The latter study [22], examined a small voxel in the pgACC in an antipsychotic naïve cohort who had no history of substance use and reported lower glutamate levels in non-responders to risperidone, although this was not significant when glutamate was scaled to the internal reference of creatine-containing metabolites. One difference between our current and previous [18] studies and the two studies that did not find an association between ACC glutamate metabolites and response in FEP [20, 44] is that we examined a more rostral region of the ACC, corresponding to the perigenual ACC (pgACC). The pgACC is involved in internal mental processes and processing affective information [45, 46]. Glutamate concentrations are higher in rostral than caudal ACC regions [47] and differentially relate with whole brain resting state functional connectivity [48]. Early changes in pgACC connectivity predict antipsychotic response in FEP [30] and pgACC glutamate correlates with ACC control over sensory regions in antipsychotic-responsive but not treatment resistant schizophrenia [49]. Therefore, one possible explanation is that the mechanisms underlying antipsychotic response are localised to within the rostral, pgACC region.

Compared to some earlier research, our current study was designed to better reflect the possible ‘real-world’ conditions under which neuroimaging and other predictive biomarkers of response might be applied soon after first presentation. Prospective studies examining glutamate metabolites in relation to response in FEP have included participants who were antipsychotic naïve or had received minimal (< 2 weeks) antipsychotic exposure [18–20, 22, 44] and most examined a single antipsychotic compound [18–20, 22], whereas in our current study all participants were medicated at the baseline scan and antipsychotics were prescribed as usual by the treating clinical team.

Unlike in the ACC, we did not observe an association between caudate glutamate metabolites and subsequent antipsychotic response. Caudate glutamate metabolites also do not appear to differ in relation to response in cross sectional studies [12, 13, 15, 16]. One study reported higher striatal glutamate longitudinally in non-responders to risperidone, although this group difference was more apparent after risperidone treatment [19]. In meta-analysis, increases in glutamate metabolites in the striatum in schizophrenia are the most consistent finding across illness stages [16, 50], which indicates that they may be less sensitive to clinical variables and thus could provide a trait marker for schizophrenia spectrum disorders [16].

In contrast to some previous studies, including those in antipsychotic naïve/minimally medicated psychosis [18, 26, 27], we did not observe decreases in glutamate metabolites during antipsychotic treatment. Although systematic review and meta-analysis has also indicated ACC or striatal glutamate metabolite decreases related to antipsychotic treatment [16, 17, 23, 24] this is below significance in several individual studies [19–21, 51–54]. The relatively short observation period and inclusion of previously medicated patients may also contribute to why we did not observe these effects. The effects of antipsychotics on glutamate are likely to be indirect and mediated through mechanisms downstream of antipsychotic binding at D2/3 and other receptor subtypes. These indirect mechanisms may lead to more subtle changes, and more variability in effect related to differences in antipsychotic pharmacology [55], or other factors such as duration of administration or patient characteristics. Glutamatergic excess associated with antipsychotic non-response is more likely to be effectively reduced by novel therapeutics which directly target the glutamate system.

While our study was designed to provide greater generalisability to normal clinical care of FEP than prior studies, the presence of antipsychotic medication at baseline and lack of standardization of antipsychotic treatment may have added variability and reduced statistical power. The presence of antipsychotic medication at baseline limits comparison to previous studies that have enrolled antipsychotic-naïve participants [19, 20, 22] and prohibits inference about relationships between glutamate and clinical outcome in the absence of antipsychotic effects. The study design, requiring capacity to consent and willingness to participate in multiple MRI scans and clinical assessments, may have excluded more severely unwell patients and this group could also be less likely to respond to antipsychotic treatment. Data acquisition and analysis was harmonized across two sites, but the application of MRI platforms from different manufacturers was associated with site effects and may have reduced statistical power. Further discussion of ^1^H-MRS site effects and standardisation is available in Egerton et al. [12]. Since the start of this study, consensus recommendations have advocated the use of semi-adiabatic localisation by adiabatic selective refocusing (semi-LASER) over PRESS for ^1^H-MRS at 3 Tesla [56]. The ^1^H-MRS signal reflects the total amount of MR-visible glutamate in the voxel rather than glutamate neurotransmission specifically. Future studies examining changes in glutamate occurring in response to stimuli with functional ^1^H-MRS [57, 58] or combining ^1^H-MRS glutamate measures with functional connectivity [49], may provide greater mechanistic information about the role of glutamate in antipsychotic response and possibly greater predictive accuracy.

Our findings have potential clinical applications. Previous research has shown that when patients fail to respond to a first antipsychotic drug they are unlikely to respond to a second non-clozapine antipsychotic [59], and that the trial of a second antipsychotic rarely results in response, and merely introduces a delay in the use of clozapine, which in turn is associated with worse outcomes [6]. Accurate prediction of non-response during initial antipsychotic treatment could therefore identify a stratum of patients most likely to benefit from clozapine as a second line treatment. Hypothetical cost-effectiveness analysis indicates that predictive models with even modest sensitivity and specificity (over 60%) to indicate clozapine as a second line antipsychotic could achieve improvements in quality of life [8]. Baseline ACC glutamate or Glx was associated with values within this range, achieving up to 79% specificity and 67% sensitivity when the early percentage change in PANSS was included in the model. However, similarly to our previous study [18], antipsychotic responders were identified more accurately (maximum PPV: 82%) than non-responders (maximum NPV: 62%). This could be related to greater clinical or biological heterogeneity within the non-responder group, for example in some cases response may have emerged over a longer observation period. For the potential application of supporting earlier clozapine initiation in non-responders, improving the NPV is of clinical importance to avoid unnecessary clozapine initiation and the associated risks. Application would also require further optimization and standardization of ^1^H-MRS data acquisition, analysis and test-retest reliability across scanner manufacturers. Gains in accuracy could also potentially be achieved by combining glutamate measures with other predictors of response, such as resting state activity [60], brain volumes [9], perfusion [61], striatal dopamine synthesis capacity [62], cognitive performance [63] and clinical or demographic predictors [41, 42, 64].

In summary, this prospective study in FEP found that higher levels of glutamate metabolites in the ACC were predictive of non-response to antipsychotic treatment 6 weeks later. This finding supports the proposition that elevated ACC metabolites may be associated with a poor antipsychotic response. Combining ^1^H-MRS glutamate measures with other variables associated with antipsychotic response may increase predictive accuracy.

## Supplementary information

Supplemental Material

## Data Availability

At the time of submission, the data governance frameworks are being put in place to make a fully anonymized version of the data available to the wider research community. To apply for access to the data, please contact J.H.M. at james.maccabe@kcl.ac.uk
