# Supplementary material for: Anterior cingulate glutamate metabolites as a predictor of antipsychotic response in first episode psychosis: data from the STRATA collaboration
Source: Neuropsychopharmacology. 2022 Dec 1;48(3):567–75. doi: 10.1038/s41386-022-01508-w (PMC9852590; doi:10.1038/s41386-022-01508-w)
Supplement: Supplementary file 1 — Supplemental Material [file 41386_2022_1508_MOESM1_ESM.pdf]

Supplementary Information: Anterior Cingulate Glutamatergic Metabolites as a Predictor of Antipsychotic Response in First Episode Psychosis.

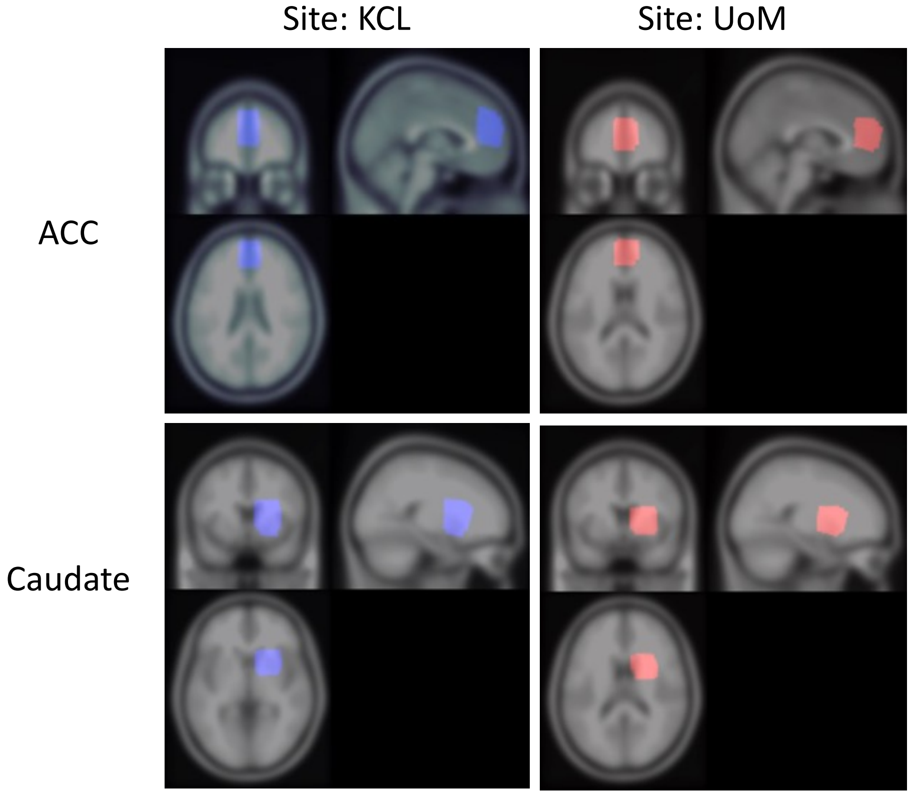

Supplement Figure 1. Images showing ^{1}H-MRS voxel placement in the anterior cingulate cortex (ACC, top row) and right caudate (bottom row), at the King's College London (KCL, left) and University of Manchester (UoM, right) sites. The images show overlaid voxels (each 20 x 20 x 20 mm^3) from study participants at baseline.

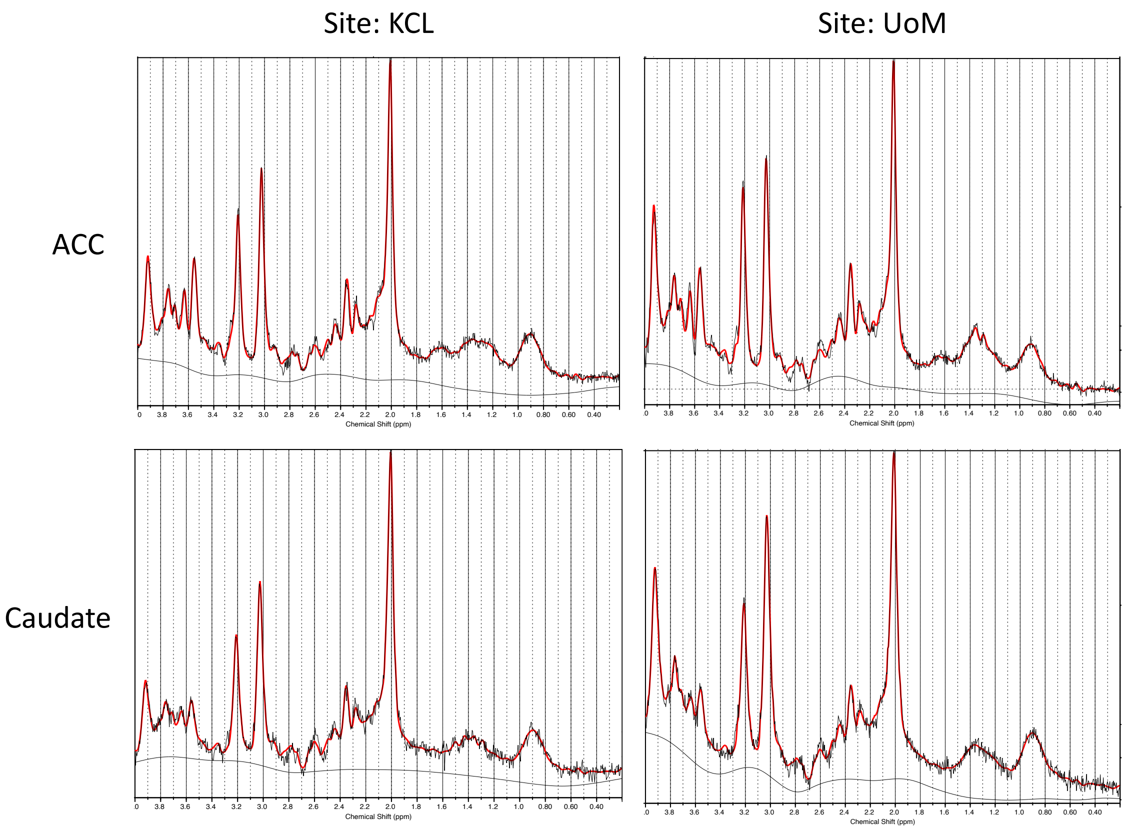

Supplement Figure 2. Example ^{1}H-MRS spectra in the anterior cingulate cortex (ACC, top row) and right caudate (bottom row), at the King's College London (KCL, left) and University of Manchester (UoM, right) sites. The figures show LCModel output of the fit in red, overlaid on the acquired spectrum (black). The estimated baseline is shown underneath in black.

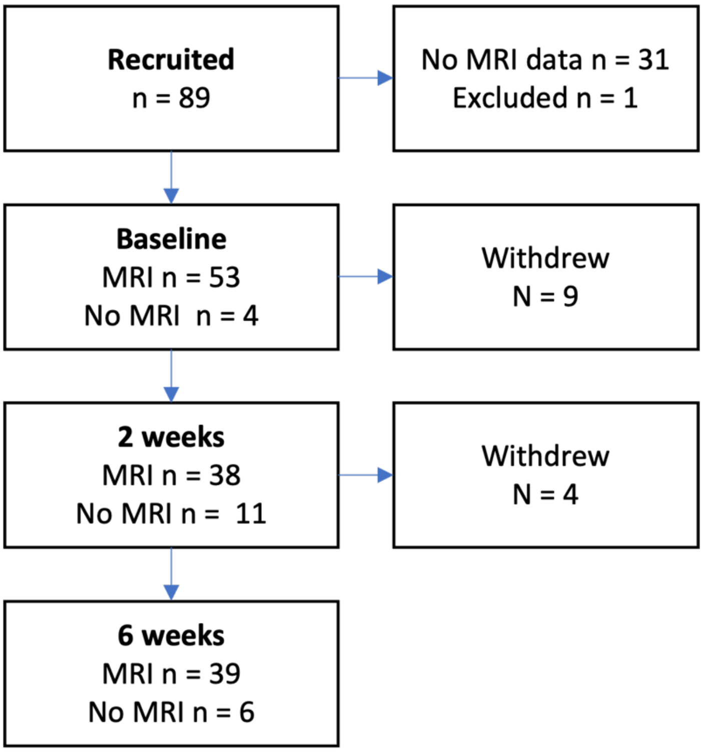

Supplement Figure 3. Participant flow though the study.

Site (Name or Number)	King’s College London	University of Manchester
1. Hardware		
a. Field strength [T]	3 T	3 T
b. Manufacturer	General Electric	Philips Achieva TX
c. Model (software version if available)	MR750	
d. RF coils: nuclei (transmit/ receive), number of channels, type, body part	C-GE HNS	Sense 32 Channel Headcoil
e. Additional hardware	None	None
2. Acquisition		
a. Pulse sequence	PRESS	PRESS
b. Volume of Interest (VOI) locations	Anterior cingulate cortex<br>Right Caudate<br>(Supplement Fig 1)	Anterior cingulate cortex<br>Right Caudate<br>(Supplement Fig 1)
c. Nominal VOI size [cm^3, mm^3]	20 x 20 x 20mm	20 x 20 x 20mm
d. Repetition Time (TR), Echo Time (TE) [ms, s]	TR = 2000ms;<br>TE = 35ms	TR = 2000ms;<br>TE = 35ms
e. Total number of Excitations or acquisitions per spectrum<br>In time series for kinetic studies<br>i. Number of Averaged spectra (NA) per time-point<br>ii. Averaging method (e.g. block-wise or moving average)<br>iii. Total number of spectra (acquired / in time-series)	128 averages	128 averages
f. Additional sequence parameters<br>(spectral width in Hz, number of spectral points, frequency offsets)<br>If STEAM:, Mixing Time (TM)<br>If MRSI: 2D or 3D, FOV in all directions, matrix size, acceleration factors, sampling method	5000 Hz; 4096 complex points.	5000 Hz; 4096 complex points.
g. Water Suppression Method	CHESS	Excitation, window = 140 Hz
h. Shimming Method, reference peak, and thresholds for “acceptance of shim” chosen	Automated B0 field mapping	Automated B0 field mapping
i. Triggering or motion correction method<br>(respiratory, peripheral, cardiac triggering, incl. device used and delays)	None	None
3. Data analysis methods and outputs		
a. Analysis software	LCModel version 6.3-1M	LCModel version 6.3-1M
b. Processing steps deviating from quoted reference or product	None	None

c. Output measure<br>(e.g. absolute concentration, institutional units, ratio)<br>Processing steps deviating from quoted reference or product	Metabolite concentration in institutional units, corrected for voxel tissue fractions	Metabolite concentration in institutional units, corrected for voxel tissue fractions
d. Quantification references and assumptions, fitting model assumptions	LCModel basis set 3t_press_te35_01a	LCModel basis set 3t_press_te35_01a
4. Data Quality		
a. Reported variables<br>(SNR, Linewidth (with reference peaks))	SNR and Linewidth, Supplement Table 3	SNR and Linewidth, Supplement Table 3
b. Data exclusion criteria	Linewidth > 2 standard deviations above mean; SNR < 2 standard deviations below mean; metabolite CRLB > 20%	Absence of corresponding unsuppressed water acquisition; Linewidth > 2 standard deviations above mean; SNR < 2 standard deviations below mean; metabolite CRLB > 20%
c. Quality measures of postprocessing Model fitting (e.g. CRLB, goodness of fit, SD of residual)	CRLB, Supplement Table 3	CRLB, Supplement Table 3
d. Sample Spectrum	Supplement Figure 2	Supplement Figure 2

Supplementary Table 1: ^{1}H-MRS details.

	Baseline	2 weeks	6 weeks
**Anterior cingulate cortex**			
**Sample size total**<br>KCL<br>UoM	**53**<br>37<br>16	**38**<br>25<br>13	**39**<br>23<br>16
**Glu_{corr}**<br>KCL<br>UoM	 22.28 ± 2.82<br>13.61 ± 1.08	 21.37 ± 2.48<br>13.75 ± 1.02	 22.06 ± 2.95<br>13.70 ± 1.33
**Glx_{corr}**<br>KCL<br>UoM	 29.59 ± 3.22<br>18.73 ± 2.04	 28.74 ± 3.86<br>18.55 ± 1.88	 28.78 ± 4.22<br>18.85 ± 1.52
**NAA_{corr}**<br>KCL<br>UoM	 18.50 ± 1.81<br>11.52 ± 0.66	 18.16 ± 3.56<br>11.15 ± 0.75	 18.94 ± 3.56<br>10.88 ± 0.62
**Cho_{corr}**<br>KCL<br>UoM	 3.84 ± 0.62<br>2.22 ± 0.36	 3.71 ± 0.66<br>2.36 ± 0.32	 4.90 ± 5.86<br>2.34 ± 0.28
**MI_{corr}**<br>KCL<br>UoM	 11.11 ± 2.01<br>7.06 ± 0.86	 10.54 ± 1.65<br>7.02 ± 0.73	 11.08 ± 2.26<br>6.98 ± 0.73
**Cr_{corr}**<br>KCL<br>UoM	 13.64 ± 1.79<br>9.51 ± 0.66	 13.31 ± 1.61<br>9.56 ± 0.86	 13.54 ± 1.73<br>9.50 ± 0.94
**Right Caudate**			
**Sample size total**<br>KCL<br>UoM	45<br>29<br>16	36<br>23<br>13	36<br>21<br>15
**Glu_{corr}**<br>KCL<br>UoM	 11.31 ± 1.48<br>8.58 ± 0.87	 11.10 ± 1.47<br>8.71 ± 1.23	 11.38 ± 1.86<br>8.53 ± 0.99
**Glx_{corr}**<br>KCL<br>UoM	 15.60 ± 2.66<br>12.70 ± 1.32	 15.04 ± 2.05<br>13.10 ± 2.36	 15.11 ± 2.81<br>12.81 ± 1.69
**NAA_{corr}**<br>KCL<br>UoM	 11.35 ± 1.24<br>7.78 ± 0.57	 11.55 ± 0.98<br>7.74 ± 0.40	 11.34 ± 0.80<br>7.89 ± 0.36
**Cho_{corr}**<br>KCL<br>UoM	 2.50 ± 1.48<br>1.77 ± 0.28	 2.31 ± 0.28<br>1.80 ± 0.23	 2.58 ± 1.48<br>1.81 ± 0.19
**MI_{corr}**<br>KCL<br>UoM	 4.40 ± 0.84<br>3.32 ± 0.85	 4.25 ± 0.73<br>3.50 ± 0.66	 4.07 ± 0.68<br>3.54 ± 0.36
**Cr_{corr}**<br>KCL<br>UoM	 8.75 ± 1.01<br>7.60 ± 0.75	 8.78 ± 0.68<br>7.23 ± 1.24	 8.70 ± 0.93<br>7.71 ± 0.66

Supplementary Table 2: ^{1}H-MRS data at each timepoint by study site. All values are corrected for voxel tissue content (Mcorr). Data are expressed as mean ± standard deviation. Glu: glutamate; Glx: glutamate plus glutamine; NAA: N-acetylaspartate plus N-acetylaspartylglutamate; MI: myo-inositol; Cho: choline; Cr: creatine.

	Baseline	2 weeks	6 weeks
**Anterior cingulate cortex**			
**Linewidth**			
KCL	0.04 ± 0.01	0.04 ± 0.01	0.04 ± 0.01
UoM	0.03 ± 0.01	0.04 ± 0.01	0.03 ± 0.01
**SNR**			
KCL	28 ± 3	27 ± 3	28 ± 3
UoM	29 ± 2	28 ± 2	29 ± 3
**Grey matter**			
KCL	0.61 ± 0.07	0.61 ± 0.05	0.61 ± 0.06
UoM	0.69 ± 0.04	0.68 ± 0.04	0.68 ± 0.05
**White matter**			
KCL	0.07 ± 0.02	0.07 ± 0.02	0.07 ± 0.02
UoM	0.08 ± 0.04	0.09 ± 0.04	0.10 ± 0.03
**CSF**			
KCL	0.32 ± 0.60	0.33 ± 0.05	0.32 ± 0.06
UoM	0.23 ± 0.03	0.23 ± 0.03	0.22 ± 0.05
**Glu CRLB**			
KCL	5 ± 1	6 ± 1	5 ± 1
UoM	5 ± 1	5 ± 1	5 ± 1
**Glx CRLB**			
KCL	6 ± 1	6 ± 1	6 ± 1
UoM	5 ± 1	5 ± 1	5 ± 1
**NAA CRLB**			
KCL	2 ± 1	3 ± 1	3 ± 1
UoM	2 ± 0	3 ± 1	2 ± 0
**Cho CRLB**			
KCL	3 ± 0	3 ± 1	3 ± 1
UoM	3 ± 1	3 ± 1	3 ± 0
**MI CRLB**			
KCL	5 ± 1	5 ± 1	5 ± 1
UoM	4 ± 1	4 ± 1	4 ± 1
**Cr CRLB**			
KCL	2 ± 0	2 ± 0	2 ± 0
UoM	2 ± 0	2 ± 1	2 ± 0
**Right Caudate**			
**Linewidth**			
KCL	0.06 ± 0.01	0.06 ± 0.01	0.06 ± 0.01
UoM	0.06 ± 0.03	0.05 ± 0.01	0.05 ± 0.01
**SNR**			
KCL	21 ± 4	21 ± 3	21 ± 3
UoM	21 ± 4	21 ± 4	21 ± 3
**Grey matter**			
KCL	0.51 ± 0.06	0.50 ± 0.05	0.52 ± 0.05
UoM	0.55 ± 0.05	0.45 ± 0.07	0.53 ± 0.06
**White matter**			
KCL	0.47 ± 0.05	0.48 ± 0.05	0.47 ± 0.05
UoM	0.41 ± 0.07	0.45 ± 0.07	0.44 ± 0.06
**CSF**			
KCL	0.02 ± 0.02	0.02 ± 0.02	0.02 ± 0.02
UoM	0.03 ± 0.05	0.03 ± 0.05	0.03 ± 0.04
**Glu CRLB**			
KCL	7 ± 1	7 ± 1	7 ± 2

UoM	7 ± 1	6 ± 1	6 ± 2
**Glx CRLB**			
KCL	8 ± 2	9 ± 2	9 ± 2
UoM	8 ± 2	7 ± 2	7 ± 1
**NAA CRLB**			
KCL	3 ± 1	3 ± 1	3 ± 1
UoM	3 ± 1	3 ± 1	3 ± 1
**Cho CRLB**			
KCL	4 ± 1	3 ± 1	3 ± 1
UoM	3 ± 1	3 ± 0	3 ± 0
**MI CRLB**			
KCL	8 ± 2	8 ± 2	9 ± 2
UoM	8 ± 3	7 ± 2	6 ± 2
**Cr CRLB**			
KCL	3 ± 1	3 ± 1	3 ± 1
UoM	2 ± 0	2 ± 0	2± 0

Supplementary Table 3: ^{1}H-MRS data quality measures at each timepoint by study site.
Data are expressed as mean ± standard deviation. FWHM: full width at half maximum linewidth (ppm); SNR: signal to noise ratio; Grey matter: voxel grey matter fraction; White matter: voxel white matter fraction; CSF: voxel cerebrospinal fluid fraction; CRLB: Cramer Rao Lower Bounds (%); Glu: glutamate; Glx: glutamate plus glutamine; NAA: N-acetylaspartate plus N-acetylaspartylglutamate; MI: myo-inositol; Cho: choline; Cr: creatine.

		**Baseline**	**2 weeks**	**6 weeks**	**Time**	**Response**	**Response x Time**
**ACC Glu_{corr}**	KCL; Responder	21.26 ± 2.53 (19)	21.43 ± 1.97 (17)	21.77 ± 2.32 (16)	$F_{2,45.34}$ = 1.34; *P* = 0.27	$F_{1,44.81}$ = 1.66; *P* = 0.20	$F_{2,45.20}$ = 2.27; *P* = 0.12
	KCL: Non-Responder	23.66 ± 2.64 (10)	21.08 ± 2.21 (6)	22.73 ± 4.20 (7)			
	UoM: Responder	13.21 ± 1.21 (6)	13.81 ± 1.27 (5)	14.12 ± 1.21 (8)			
	UoM: Non-Responder	13.89 ± 1.03 (7)	13.52 ± 0.79 (7)	13.14 ± 1.37 (8)			
**ACC Glx_{corr}**	KCL; Responder	27.96 ± 2.50 (19)	29.14 ± 3.34 (17)	28.54 ± 4.57 (16)	$F_{2,41.44}$ = 0.45; *P* = 0.64	$F_{1,45.40}$ = 2.79; *P* = 0.10	$F_{2,41.39}$ = 3.29; *P* = 0.05
	KCL: Non-Responder	31.37 + 3.54 (10)	27.95 ± 2.74 (6)	29.34 ± 3.55 (7)			
	UoM: Responder	17.84 ± 1.53 (6)	18.07 ± 2.23 (5)	18.75 ± 1.25 (8)			
	UoM: Non-Responder	19.39 ± 2.52 (7)	18.69 ± 1.80 (7)	18.94 ± 1.84 (8)			

**Caudate Glu_{corr}**	KCL; Responder	11.30 ± 1.06 (15)	11.38 ± 1.41 (16)	11.70 ± 1.30 (15)	$F_{2;39.96}$ = 0.31; *P* = 0.73	$F_{1,37.13}$ = 3.75; *P* = 0.06	$F_{2,39.95}$ = 0.63; *P* = 0.54
	KCL: Non-Responder	11.09 ± 2.33 (9)	10.21 ± 1.50 (6)	10.58 ± 2.84 (6)			
	UoM: Responder	8.75 ± 0.59 (6)	8.76 ± 1.78 (6)	8.80 ± 1.06 (7)			
	UoM: Non-Responder	8.57 ± 1.15 (7)	8.75 ± 0.66 (6)	8.30 ± 0.92 (8)			
**Caudate Glx_{corr}**	KCL; Responder	15.18 ± 2.53 (15)	15.40 ± 1.90 (16)	15.76 ± 1.78 (15)	$F_{2;37.13}$ = 0.34; *P* = 0.72	$F_{1,51.93}$ = 2.76; *P* = 0.10	$F_{2,37.12}$ = 1.31; *P* = 0.28
	KCL: Non-Responder	15.85 ± 3.45 (9)	13.63 ± 2.25 (5)	13.46 ± 4.27 (6)			
	UoM: Responder	13.14 ± 1.23 (6)	13.05 ± 3.25 (6)	13.37 ± 1.86 (7)			
	UoM: Non-Responder	12.66 ± 1.37 (7)	13.16 ± 1.67 (6)	12.32 ± 1.48 (8)			

Supplement Table 4. Glutamate metabolites in the caudate and ACC at each assessment visit, by 6-week response status and by recruitment site. KCL: King's College London; UoM: University of Manchester. Glutamate metabolites are presented as mean ± standard deviation (sample size). The table presents the results of the linear mixed model analyses.
